# Synergistic Effects of Vascular Endothelial Growth Factor on Bone Morphogenetic Proteins Induced Bone Formation In Vivo: Influencing Factors and Future Research Directions

**DOI:** 10.1155/2016/2869572

**Published:** 2016-12-13

**Authors:** Bo Li, Hai Wang, Guixing Qiu, Xinlin Su, Zhihong Wu

**Affiliations:** ^1^Department of Orthopaedic Surgery, Peking Union Medical College Hospital, Peking Union Medical College and Chinese Academy of Medical Sciences, No. 1 Shuaifuyuan, Beijing 100730, China; ^2^Central Laboratory, Peking Union Medical College Hospital, Peking Union Medical College and Chinese Academy of Medical Sciences, No. 1 Shuaifuyuan, Beijing 100730, China; ^3^Beijing Key Laboratory for Genetic Research of Bone and Joint Disease, No. 1 Shuaifuyuan, Beijing 100730, China

## Abstract

Vascular endothelial growth factor (VEGF) and bone morphogenetic proteins (BMPs), as key mediators in angiogenesis and osteogenesis, are used in a combined delivery manner as a novel strategy in bone tissue engineering. VEGF has the potential to enhance BMPs induced bone formation. Both gene delivery and material-based delivery systems were incorporated in previous studies to investigate the synergistic effects of VEGF and BMPs. However, their results were controversial due to variation of methods incorporated in different studies. Factors influencing the synergistic effects of VEGF on BMPs induced bone formation were identified and analyzed in this review to reduce confusion on this issue. The potential mechanisms and directions of future studies were also proposed here. Further investigating mechanisms of the synergistic effects and optimizing these influencing factors will help to generate more effective bone regeneration.

## 1. Introduction

Globally, approximately 15 million fractures are reported per year [1, 2], with 5–10% nonunion rate [1, 3, 4]. In addition, other diseases including tumors, infections, and degenerative diseases may also lead to bone defect. Bone is the second most commonly transplanted tissue, preceded only by blood transfusion [1]. Bone grafts performed in the United States alone are approximately 1.6 million per year, bringing huge medical and economic burden [2]. A variety of strategies have been developed to repair the diseased or defected bone. Autografts, which can provide desired growth factors, cells, and even microcirculation system, have always been regarded as the gold standard [3, 5, 6]. Unfortunately, autografts are restricted by some disadvantages, such as limited donor availability and donor-site morbidity [3, 6]. Allografts and xenografts as alternatives are limited by the potential disease transmission and immune rejection [3, 6, 7]. Tissue engineering is generally considered as a promising technique to overcome the disadvantages of traditional therapies.

A major objective of bone tissue engineering is to get effective bone regeneration, which is related to successful osteoinduction. Osteogenic cytokines, such as bone morphogenetic proteins (BMPs) and platelet-derived growth factor (PDGF), are the ideal candidates to enhance the osteoinduction [8, 9]. BMPs, identified and named by Urist [10, 11], belong to transforming growth factors *β* (TGF-*β*) superfamily. The osteogenic ability of BMPs has been well-documented in literatures [12]. Among them, BMP2, BMP4, BMP6, BMP7, and BMP9 possess osteogenic properties [12, 13]. Furthermore, rhBMP2 and rhBMP7 have been approved by FDA (US Food and Drug Administration) for specific clinical applications [12, 14]. However, in the last few years, some studies showed that desired clinical results could not been obtained by using BMPs alone. Moreover, complications caused by high-dose application were worrisome [3, 15–18].

Blood supply is arguably the largest challenge for any tissue engineering [25, 26]. Within the body, the effective diffusion distance of oxygen and nutrients is no more than 200 *μ*m from the nearest capillary [25–27]. Bone is a highly vascularized tissue. Reconstructing local microcirculation is prerequisite for effective bone regeneration [28]. Inhibiting angiogenesis will reduce bone formation [29–31], while promoting angiogenesis can enhance bone regeneration [32]. Angiogenesis is regulated by several angiogenic factors, such as fibroblast growth factors (FGFs), transforming growth factor-*α*/*β* (TGF-*α*/*β*), PDGF, and notably vascular endothelial growth factor (VEGF) [15, 33–37]. Gerber et al. [29] investigated the role of VEGF in angiogenesis and bone formation. Their results shown that vascular invasion and bone formation were both suppressed by inhibiting VEGF in 24-day-old mice [29]. It was also revealed that application of VEGF-specific antagonist (soluble Flt1) could inhibit the bone regeneration induced by BMP4 and BMP2 [19, 20].

The undesirable outcomes of using BMPs alone and the importance of blood supply inspire tissue engineering scientists to explore the combined application of osteogenic and angiogenic factors [15, 38–40]. One of the most studied directions is the codelivery of BMPs and VEGF. The addition of VEGF is expected to enhance bone formation and reduce the amount of BMPs used.

## 2. The Combined Application of VEGF and BMPS

### 2.1. The Role of VEGF on BMPs Induced Bone Formation

**Figure 1:**
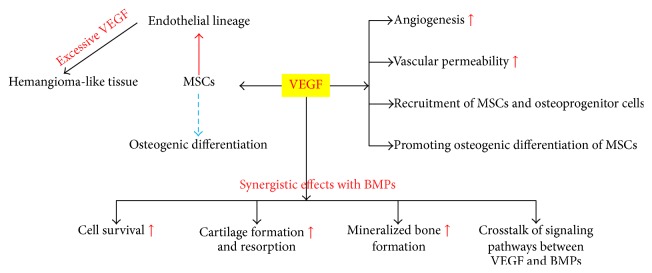
The role of VEGF on BMPs induced bone formation.

As a key mediator of angiogenesis [15, 38], VEGF also has direct and indirect effects on bone formation [7, 43]. VEGF may increase vascular permeability after promoting local angiogenesis [43, 49]. This will facilitate the recruitment of mesenchymal stem cells (MSCs) and osteoprogenitor cells to indirectly enhance the ability of bone regeneration [7, 43]. VEGF can also directly attract MSCs and promote their osteogenic differentiation [7, 43]. Enhanced neovascularization and bone regeneration were induced by the controlled release of VEGF in the study of Kaigler et al. [32]. After blocking VEGF, angiogenesis and osteogenesis were both inhibited [19, 20]. In addition to increasing angiogenesis and recruitment of MSCs, VEGF can act synergistically with BMPs to enhance cell survival, cartilage formation and resorption, and mineralized bone formation [19, 20]. Recently, the cross-talk of signaling pathways between VEGF and BMPs has gained growing attention [15, 50, 51]. Studies indicated that the synergistic effects of VEGF on BMPs induced bone formation were not only due to the increased angiogenesis (Figure 1). After the activation of VEGF signaling, the response of MSCs to BMP6 was significantly enhanced both in vitro and in vivo [15, 23, 51]. When treated with VEGF and BMP6, the expression of osteogenic genes including ALP, Dlx5, and osterix was significantly upregulated [50]. Furthermore, BMP-nonresponsive osteoprogenitor cells responded well to the costimulation of VEGF and BMP6 [51]. However, the accurate mechanisms are still unknown.

### 2.2. Controversy on the Synergistic Effects between VEGF and BMPs

**Figure 2:**
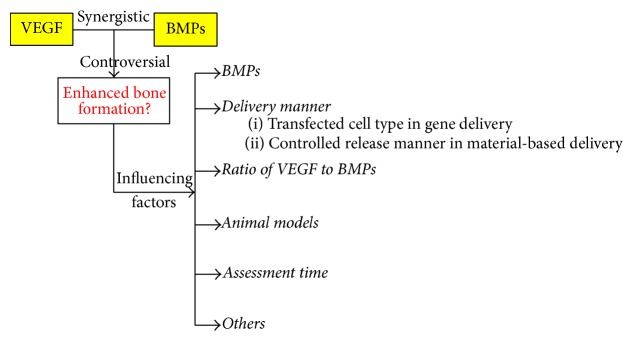
Factors influencing the synergistic effects between VEGF and BMPs.

Although many studies have focused on this issue, whether VEGF can enhance BMPs induced bone formation in vivo is still very controversial [46]. The variation of influencing factors in different studies has led to completely opposite results, bringing much confusion on this issue. Based on literatures in this field, several important factors (Figure 2), which can significantly influence the synergistic effects between VEGF and BMPs, were identified and analyzed in the present review. These factors could partly explain the variations in the results of different studies and provide important information for future studies to generate more effective bone regeneration.

## 3. Influencing Factors 

### 3.1. BMPs

Among BMP family, BMP2, BMP4, BMP6, BMP7, and BMP9 possess osteogenic properties [12, 13, 52–54]. They may share some properties in osteoinductive activity, but their interactions with VEGF are distinct. Synergistic effect of BMP4 with VEGF on bone formation is quite different from that of BMP2. Osteogenic effect of BMP4 was significantly affected by exogenous VEGF and it was more sensitive to the ratio of VEGF to BMP4 [19, 20]. High ratio of VEGF/BMP4 was obviously detrimental to the mineralized bone formation [19]. However, BMP2 could induced well-formed mineralized bone under high ratio of VEGF/BMP2, although the amount of bone formation also decreased compared to the group with lower ratio of VEGF/BMP2 [20]. The reason why BMP2 is less sensitive to VEGF remains unclear. A possible explanation is that BMP2 itself possesses angiogenic activity, leading to a decreased reaction to VEGF [20].

### 3.2. Delivery Manner of VEGF and BMPs

Traditional administration of growth factors is limited by their relatively short half-lives and potential side effects [55–57]. To overcome these disadvantages, gene delivery [6, 58–63] and material-based delivery system [64–70] have been developed in tissue engineering. There are mainly two strategies used in the codelivery of VEGF and BMPs. One of them is the expression of transgenes [19, 71–75] and the other is controlled release of growth factors from specific materials [38, 67, 76–78]. Transfected cell types in gene delivery and controlled release manners in material-based delivery can obviously influence the synergistic effects of VEGF and BMPs.

#### 3.2.1. Transfected Cell Types in Gene Delivery

For transgenes therapy, plasmid, virus, and transfected cells are usually used as vectors or carriers for sustained expression of VEGF and/or BMPs [19–24, 79, 80] (Table 1). When transfected cells are transplanted in vivo, the synergistic effects of VEGF on BMPs induced bone formation are cell-type dependent. Peng et al. [19] transfected muscle-derived stem cells (MDSCs) to express VEGF or BMP4. Combined transplantation of VEGF- and BMP4-expressing cells resulted in significantly more bone formation compared to transplantation of BMP4-expressing cells alone [19]. Human periosteum-derived cells, osteoprogenitor cells, and bone marrow stromal cells (BMSCs) have been also proven to be effective carriers to achieve the synergistic effects between VEGF and BMPs [21, 23, 79]. However, when C2C12 cells (mouse myoblasts) and NIH/3T3 cells (mouse fibroblasts) were transfected to express BMP4 or VEGF + BMP4, VEGF inhibited the calcification of cells in vitro and exhibited a detrimental effect on bone formation in vivo [22].

#### 3.2.2. Controlled Release Manners in Material-Based Delivery

Studies have adopted different controlled release manners for delivering VEGF and BMPs to investigate their synergistic effects [7, 13, 41–48, 81–83] (Table 2). Biomaterials, such as gelatin, chitosan, collagen and poly (lactic-co-glycolic acid) (PLGA), can serve as carriers to release growth factors in a sustained manner in vivo [38, 84–87]. The controlled drug delivery system can be incorporated into porous materials to form a hybrid bone substitute scaffold, which can fill bone defect and induce effective bone repair. During normal bone regeneration, the expression of VEGF is upregulated in the early days and peaks around day 5–10 [43, 88–90], while normal expression of BMPs peaks at day 21 and thereafter [43, 91, 92]. In order to achieve the sequential release of growth factors, Kempen et al. [43] adopted PLGA microspheres and poly (propylene) scaffold as a sustained release system of BMP2 and used gelatin hydrogel as a fast release system of VEGF. The in vivo release profiles of VEGF showed an initial burst release in the first 3 days (89.9 ± 2.9% at the ectopic site). The remaining VEGF exhibited a sustained release over 35 days at a low level. The release of BMP2 was sustained over 56 days [43]. Ectopic bone formation was significantly enhanced by the combined application of VEGF and BMP2 compared to that of BMP2 alone [43]. However, some other authors supported more sustained delivery (nonfast release) of VEGF [7, 32]. The effect of fast release of VEGF is still controversial. The vascular network induced by VEGF alone is immature [26, 93–95]. If its concentration falls too low before the formation of mature vascular network, the unstable vascular network may be remodeled or trimmed [42, 95, 96]. This is also supported by the results of previous studies that synergistic effect of VEGF and BMP2 was only presented at 4 weeks in vivo, while being absent at 12 weeks [42]. In both groups of VEGF alone and VEGF + BMP2, a decrease of vascular density was observed at 12 weeks compared to that at 4 weeks [46]. In fact the effective delivery manner of VEGF in the study of Kempen et al. [43] is more like a composite model: burst release in the early stage and sustained delivery at a low level in later stage. The optimal delivery manner of VEGF needs to be further studied. In addition, combining other angiogenic factors, such as PDGF, to facilitate the maturity and stability of neovascularization may be more effective [95].

Although some authors reported that fast release of BMP2 might induce more ectopic bone regeneration than its sustained release [13], more papers confirmed that the sustained delivery strategy would prolong its activity and reduce its potential side effects [7, 43, 97–101].

### 3.3. Ratio of VEGF to BMPs

The ratio of VEGF to BMPs has an obvious impact on their synergistic effects. Although the interactions between BMPs and VEGF are inconsistent among different kinds of BMPs, there is a similar trend that VEGF seems to be more effective at low ratio of VEGF/BMPs than at a high ratio [19, 20]. Peng et al. [19] have adopted the cotransplantation of VEGF- and BMP4-expressing muscle-derived stem cells at different ratios to study its relationship with bone formation. The amount of bone formation in groups with the ratios of VEGF/BMP4 at 1 : 5 and 1 : 1 was significantly larger compared to that in the group with a ratio of 5 : 1 [19]. The interactions of VEGF and BMPs are based on their influence on the function and differentiation of target cells. Under high ratio of VEGF/BMPs, excessive VEGF will push local MSCs towards an endothelial lineage, reducing the cells available for osteogenic differentiation [19, 44]. It was reported that high dose of VEGF might lead to hemangioma-like tissue formation [102, 103]. It was also suspected that high ratio of VEGF/BMPs may increase the recruitment and survival of osteoclasts, leading to excessive bone resorption [15, 104–106]. However, Peng et al. [19] disagreed with this. In their study, the markers of osteoclasts were similar in groups with low and high ratios of VEGF/BMP4 [19]. It is important to note that ratio of VEGF/BMPs reported in most studies is the ratio of total dose of growth factors [42, 44] or total amount of transfected cells used [19, 20]. However, what actually affects the bone formation is the ratio of released growth factors. As reported by Lohse et al. [83], continuous delivery of VEGF and BMP2 at a ratio approximately 1 could significantly increase the induced bone formation compared to that at a ratio ≤0.5. Future studies should further investigate the relationship between the ratio of released VEGF/BMPs and the amount of bone formation both in vitro and in vivo.

### 3.4. Animal Models

When other experimental conditions are controlled to be consistent, the synergistic effects of VEGF and BMPs vary among different animal models. In the same studies [13, 43], synergistic effects between VEGF and BMPs were only observed in ectopic models, while being absent in orthopaedic sites, indicating the synergistic effect was location-dependent. Facilitating the recruitment of MSCs is one of the mechanisms why VEGF can enhance bone formation elicited by BMPs. Nevertheless, in the bone defect site, periosteum and exposed marrow cavity can offer an abundant of MSCs [43]. Furthermore, local hematoma in orthopaedic site may serve as a source of endogenous angiogenic factors [48, 107–109]. The abundant source of MSCs and increased endogenous angiogenic factors may decrease the effect of exogenous VEGF. The synergistic effects between VEGF and BMPs are supposed to be more prominent in areas suffering from compromised circulation, such as ischemia model and old bone defect model.

### 3.5. Assessment Time

The synergistic effects of VEGF and BMPs might be observed in a short study period, while being absent in an extended period [42, 46]. The decrease of concentration of growth factors, such as VEGF, may partly explain this, as analyzed above. Another possibility proposed in this review is whether the application of exogenous growth factors will downregulate the secretion of endogenous VEGF and BMPs within a certain period. If so, after depletion of exogenous growth factors, the lack of endogenous growth factors will be detrimental to bone regeneration. Extending observation period and setting different time points should be helpful to get further understanding of the synergistic effects and to optimize the combination application strategies in the future studies.

### 3.6. Other Influencing Factors

In addition to the factors mentioned above, material carriers of the delivery system, methods used in assessment of bone formation, and the introduction of other growth factors or cells might also influence the evaluation of synergistic effects of VEGF and BMPs. Effective control of these related factors can help us to get further understanding of the mechanisms of the interactions between these two key growth factors in angiogenesis and osteogenesis.

## 4. Conclusions

The combined delivery of VEGF and BMPs is a novel and promising strategy in bone tissue engineering. VEGF can help to promote the construction of vascular network, to improve the local supply of oxygen and nutrients, to increase the recruitment and survival of MSCs, and to enhance the response of MSCs to BMPs. When they are used in a combined delivery manner in vivo, VEGF has the potential to synergistically enhance BMPs induced bone formation. Many studies have been conducted to investigate the effect of this approach. However, due to the variation of BMPs, carriers of growth factors, controlled release manners, growth factors ratio, models, and assessment time, their results are pretty controversial. These influencing factors were identified and analyzed in this review to avoid more confusion on this issue. Future studies should further investigate the mechanisms of their synergistic effects and optimize these influencing factors to generate more effective bone regeneration.

## Figures and Tables

**Table 1:** Gene delivery of VEGF and BMPs.

Authors	BMPs	Vectors (cell-type)	Models	Expression in combination group	Ratio (VEGF/BMP)	Bone formation in combined delivery group
Peng et al. 2002 [19]	BMP4	Retroviral vectors (MDSCs) Cell-expressing BMP4 or VEGF	Mice Intramuscular (4 weeks) Calvarial defect (3/6 weeks) Calvarial defect (3 weeks)	BMP4: 115 ± 20 ng/10^6^ cells/24 h VEGF: 214 ± 35 ng/10^6^ cells/24 h (>4 weeks)	Cell ratio: 1 : 5 1 : 5 1 : 5 (group 1, G1) 1 : 1 (group 2, G2) 5 : 1 (group 3, G3)	Enhanced (compared to BMP4 alone) Enhanced (compared to BMP4 alone) Increased (compared to G3) Increased (compared to G3) Decreased (compared to G1/2) [19]

Peng et al. 2005 [20]	BMP2	Retroviral vectors (MDSCs) Cell-expressing BMP2 or VEGF	Mice Intramuscular (4 weeks) Calvarial defect (3/6 weeks) Calvarial defect (3 weeks)	BMP2: 250 ng/10^6^ cells/24 h VEGF: 200 ng/10^6^ cells/24 h (>4 weeks)	Cell ratio: 1 : 5 1 : 5 1 : 5 (group 1, G1) 1 : 1 (group 2, G2) 5 : 1 (group 3, G3)	Enhanced (compared to BMP2 alone) Enhanced (compared to BMP2 alone) Increased (compared to G3) Increased (compared to G3) Decreased (compared to G1/2) [20]

Samee et al. 2008 [21]	BMP2	Plasmid vectors (human periosteal cells) Cell-expressing BMP2 and/or VEGF	Mice Intramuscular (4/8 weeks)	BMP2: ~600–950 ng/10^6^ cells/12–48 h VEGF: ~1.5–7.5 ng/10^6^ cells/12–48 h (within 5 weeks)	Plasmid ratio in cotransfection: 1 : 1	Enhanced at 4 weeks (compared to BMP2 alone) No significant difference at 8 weeks (compared to BMP2 alone) [21]

Li et al. 2009 [22]	BMP4	Retroviral vectors (C2C12 and NIH/3T3 cells) Cell-expressing BMP4 and/or VEGF	Mice Intramuscular (3-5weeks)	C2C12: BMP4: 105 ± 10 ng/10^6^ cells/24 h, VEGF: 152 ± 20 ng/10^6^ cells/24 h NIH/3T3: BMP4: 98 ± 5 ng/10^6^ cells/24 h VEGF: 128 ± 12 ng/10^6^ cells/24 h (Retroviral vectors ratio 1 : 1)	Retroviral vectors ratio in cotransfection: 1 : 1 1 : 5 1 : 50	Inhibited (compared to BMP4 alone) More detrimental effect Detrimental effect Less detrimental effect (compared to BMP4 alone) [22]

Cui et al. 2010 [23]	BMP6	Plasmid vectors (cloned mouse osteoprogenitor cells) Cell-expressing BMP6 and/or VEGF	Mice Subcutaneous (2/3 weeks)	BMP6: ~1.8–18 ng/10^6^ cells/24 h VEGF: ~0.12–12 ng/10^6^ cells/24 h (within 2 weeks)	VEGF/BMP6 gene (1 : 1) incorporated in plasmid constructs	Enhanced (compared to BMP6 alone) [23]

Xiao et al. 2011 [24]	BMP2	Adenoviruse vectors (rabbit bone marrow stromal cells) Cell-expressing BMP2 or VEGF	Rabbit Orbital defects (4/8/16 weeks)	BMP2: ~300 pg/10^6^ cells/24 h VEGF: ~300 pg/10^6^ cells/24 h (within 8 weeks)	Cell ratio: 1 : 4 (VEGF/BMP ratio 1 : 1)	Enhanced (compared to BMP2 alone) [24]

MDSCs = mouse muscle-derived stem cells, C2C12 cells = mouse myoblasts, and NIH/3T3 cells = mouse fibroblasts.

**Table 2:** Controlled release VEGF and BMPs.

Authors	BMPs	Carrier	Models	Combination delivery	Ratio (VEGF/BMP)	Bone formation in combined delivery group
Kakudo et al. 2006 [41]	BMP2	Collagen	Rat Intramuscular (3 weeks)	Simultaneous	1 : 2	Enhanced (compared to BMP2 alone) [41]

Patel et al. 2008 [42]	BMP2	Gelatin	Rat Calvarial defect (4/12 weeks)	Simultaneous	6 : 1	Enhanced at 4 weeks (compared to BMP2 alone) No significant difference at 12 weeks (compared to BMP2 alone) [42]

Kempen et al. 2009 [43]	BMP2	PLGA-BMP2 (sustained release) Gelatin-VEGF (fast release)	Rat Subcutaneous (8 weeks) Segmental femoral defect (8 weeks)	Sequential	1 : 3.3	Ectopic: enhanced (compared to BMP2 alone) Orthotopic: no significant difference (compared to BMP2 alone) [43]

Young et al. 2009 [44]	BMP2	Gelatin	Rat Calvarial defect (12 weeks)	Simultaneous	6/12/24 : 1	No significant difference (compared to BMP2 alone) [44]

Roldán et al. 2010 [45]	BMP7	BCP scaffold (growth factors injected in the scaffolds)	Mice Subcutaneous (12 weeks)	Simultaneous	2 : 5	No significant difference (compared to BMP7 alone) [45]

Zhang et al. 2011 [7]	BMP2	Silk hydrogels	Rabbits Sinus floor elevation model (4/12 weeks)	Simultaneous	2 : 3	Enhanced (compared to BMP2 alone) [7]

Geuze et al. 2012 [13]	BMP2	PLGA-VEGF/BMP2 (fast release) Gelatin-VEGF/BMP2 (sustained release)	Dog Ectopic: intramuscular (9 weeks) Orthotopic site: ulnar defect (9 weeks)	Sequential or simultaneous	1 : 30	No significant enhancement effect (compared to BMP2 alone) [13]

Hernández et al. 2012 [46]	BMP2	PLGA	Rabbit Intramedullary femur defect (4/12 weeks)	Simultaneous	1 : 10/50	Enhanced at 4 weeks (compared to BMP2 alone) No significant difference at 12 weeks (compared to BMP2 alone) [46]

Das et al. 2015 [47]	BMP6	PLGA	Rat Mandibular defect (2/8/12 weeks)	Simultaneous	1 : 1	Enhanced (compared to BMP6 alone) [47]

Lv et al. 2015 [48]	BMP2	Fibrin glue (fast release)	Rabbit Femoral condyle defect (4 weeks)	Simultaneous	1 : 100	No synergistic effect (compared to BMP2 alone) [48]
